# Immunisation against Measles

**Published:** 1919-06-07

**Authors:** 


### Immunisation Against Measles.

AN interesting suggestion arises from some experiments recently recorded by Drs. D. L. Richardson and Hilary Connor in the Journal of the American Medical Association. To give a brief outline, it may be explained that six children were exposed to a definite infection of measles and were apparently protected by an immune serum; one child, similarly exposed and used a control, developed the disease. Three other children received simultaneous inoculation with virus and immune serum; in two there was no reaction whatever, in the third there was a slight transient pipexia and a brief atypical rash.

The method of preparing this immune serum is described as follows: From recently recovered children blood drawn in the usual way from veins at the flexor surface of the elbow was collected in marked sterile tubes and kept overnight in the refrigerator. Next day the Wassermann test was applied to each sample separately, the sera which reacted negatively being then mixed and used for immunisation treatment. Administration was made intramuscularly into the thigh in doses up to 25 c.c., if possible within a few days of exposure to the disease. No reactions, local or general, immediate or delayed, were noted.

Virus inoculation was made by swabs rubbed gently over the nasal mucosa of infants it was desired to infect. One may say that such experiments, described in outline and limited in number, perhaps for reason of many circumstantial difficulties inevitably to be encountered in such work are, of course, too few to be conclusive, but worthy of considerable further investigation. Alhough there are two sides to a question of advisability in providing temporary immunity against this universal disease of childhood which so markedly provides its own protection against second attacks, we are certainly in need of a therapeutic agent which will constantly lessen the intensity of constitutional effects once the infection has occurred. Perhaps this small work is the forerunner of some such provision.

